# A reference high-pressure CO_2_ adsorption isotherm for ammonium ZSM-5 zeolite: results of an interlaboratory study

**DOI:** 10.1007/s10450-018-9958-x

**Published:** 2018-07-26

**Authors:** H. G. T. Nguyen, L. Espinal, R. D. van Zee, M. Thommes, B. Toman, M. S. L. Hudson, E. Mangano, S. Brandani, D. P. Broom, M. J. Benham, K. Cychosz, P. Bertier, F. Yang, B. M. Krooss, R. L. Siegelman, M. Hakuman, K. Nakai, A. D. Ebner, L. Erden, J. A. Ritter, A. Moran, O. Talu, Y. Huang, K. S. Walton, P. Billemont, G. De Weireld

**Affiliations:** 1 National Institute of Standards and Technology, Gaithersburg, MD USA; 2 Quantachrome Instruments, Boynton Beach, FL USA; 3 University of Edinburgh, Edinburgh, UK; 4 Hiden Isochema Limited, Warrington, UK; 5 RWTH Aachen University, Aachen, Germany; 6 University of California, Berkeley, CA USA; 7 MicrotracBEL, Suminoe-ku, Osaka, Japan; 8 University of South Carolina, Columbia, SC USA; 9 Cleveland State University, Cleveland, OH USA; 10 Georgia Institute of Technology, Atlanta, GA USA; 11 University of Mons, Mons, Belgium

**Keywords:** Carbon dioxide, High-pressure adsorption isotherm, Interlaboratory study, Reference adsorbent material, Reference isotherm, RM 8852, Surface excess adsorption, ZSM-5

## Abstract

This paper reports the results of an international interlaboratory study led by the National Institute of Standards and Technology (NIST) on the measurement of high-pressure surface excess carbon dioxide adsorption isotherms on NIST Reference Material RM 8852 (ammonium ZSM-5 zeolite), at 293.15 K (20 °C) from 1 kPa up to 4.5 MPa. Eleven laboratories participated in this exercise and, for the first time, high-pressure adsorption reference data are reported using a reference material. An empirical reference equation ${n_{ex}}=\frac{d}{{{{(1+\exp [\left( { - {\text{ln}}(P)+a} \right)/b~])}^{c~~}}}}~,$ [*n*_*ex*_-surface excess uptake (mmol/g), *P*-equilibrium pressure (MPa), *a* = −6.22, *b* = 1.97, *c* = 4.73, and *d* = 3.87] along with the 95% uncertainty interval (*U*_*k* = 2_ = 0.075 mmol/g) were determined for the reference isotherm using a Bayesian, Markov Chain Monte Carlo method. Together, this zeolitic reference material and the associated adsorption data provide a means for laboratories to test and validate high-pressure adsorption equipment and measurements. Recommendations are provided for measuring reliable high-pressure adsorption isotherms using this material, including activation procedures, data processing methods to determine surface excess uptake, and the appropriate equation of state to be used.

**Electronic supplementary material:**

The online version of this article (10.1007/s10450-018-9958-x) contains supplementary material, which is available to authorized users.

## Introduction

Adsorbent materials have many applications, including those related to gas storage, gas separation and purification, catalytic reforming, and environmental remediation (Dabrowski 2001; Yang 2003). To better understand and optimize the performance of adsorbents, significant effort has been invested toward adsorbent characterization, and progress has been realized during the past two decades, mainly through low-pressure cryogenic adsorption experiments (Thommes et al. 2015). During the same period, many high-pressure adsorption measurements have also been reported for fluids on micro- and mesoporous solids (Menon 1968; Findenegg and Thommes 1997; Malbrunot et al. 1997; White et al. 2005). However, challenges still exist for obtaining reliable high-pressure adsorption isotherms, as demonstrated in a series of interlaboratory studies (ILSs) on molecular hydrogen (Broom and Hirscher 2016; Hurst et al. 2016; Moretto et al. 2013; Zlotea et al. 2009), carbon dioxide (Gensterblum et al. 2009, 2010; Goodman et al. 2004, 2007; Gasparik et al. 2014) and small hydrocarbons (Gasparik et al. 2014). These challenges are associated, in part, with the lack of standardized protocols, reference materials, and reference data (Espinal et al. 2013; Broom and Webb 2017).

In response to this situation, the National Institute of Standards and Technology (NIST) partnered with the U.S. Department of Energy’s Advanced Research Projects Agency-Energy (ARPA-E) to create the Facility for Adsorbent Characterization and Testing (FACT Lab).^1^ The FACT Lab recently sponsored a workshop on “Measurement Needs in the Adsorption Sciences.” The workshop recommended that an interlaboratory study of high-pressure adsorption isotherm measurements on an existing NIST reference material be undertaken (Thommes and van Zee 2015).

For this ILS, NIST Reference Material RM 8852 (ammonium ZSM-5 zeolite) (Turner et al. 2008) was selected as the adsorbent because it consists of a network of narrow micropores (≈0.5 nm) (Kokotailo et al. 1978), is an important catalyst (Cejka et al. 2017), and is the least hygroscopic among three NIST zeolitic reference materials (RM 8850, RM 8851, and RM 8852), though RM 8852 is somewhat hygroscopic (Si/Al ≈ 28.3, loss on ignition ≈ loss on fusion ≈ 8.5%) (Turner et al. 2008). As a reference material, RM 8852 offers the advantage of having been homogenized and characterized for a wide range of physical and chemical properties. Finally, the existing stock of this material is sufficient to ensure availability to the adsorption science community for the foreseeable future.

Carbon dioxide (CO_2_) was selected as the adsorptive because of its importance in gas storage and separation applications, its thermophysical properties near ambient temperature would provide an extra test of experimental procedures, and it is a gas that most labs would be equipped to handle. Also, high-pressure CO_2_ adsorption on nanoporous materials, such as activated carbon, coal, shales, zeolites, MOFs, and mesoporous silica, has been previously studied for carbon capture and sequestration due to concern over its impact on the climate (Humayun and Tomasko 2000; Gao et al. 2004; Moellmer et al. 2010; Rother et al. 2012; Gensterblum et al. 2010; Goodman et al. 2004; Gasparik et al. 2014; Choi et al. 2009; Sumida et al. 2012; Espinal and Morreale 2012; Espinal et al. 2013; Bae and Snurr 2011; Lin et al. 2012). Reliable measurements of high-pressure CO_2_ adsorption isotherms are therefore helpful for developing design principles for new and improved solid adsorbents.

The objectives of this ILS were three-fold: (1) to provide an assessment of the comparability of high-pressure adsorption isotherms across measurement techniques and procedures, as currently practiced, (2) to generate a reference isotherm on a reference material to serve as a standard for the adsorption community, and (3) to recommend best-practices for high-pressure isotherm measurements based on the results of the exercise. An aspect that distinguishes this ILS from previous studies is that differences among submitted data were investigated and, as a collaborative effort, participants were given the opportunity to remeasure or reprocess submitted isotherms before the reference isotherm was derived.

## Experimental and data analysis methods

Ten invited laboratories participated in this ILS, in addition to the FACT Lab. The measurement capabilities of these laboratories included both commercial and custom-built manometric and gravimetric instruments.

### ILS protocol

Given that one objective was to assess existing laboratory practices, the measurement protocol was not overly detailed. It only specified a minimum purity of the CO_2_ (99.999%), the sample pretreatment protocol [activation at 623 K (350 °C) for at least 12 h under high-vacuum], the pressure range (4.5 MPa or the maximum capability of the instrument), the temperature of the isotherm (293.15 K, 20 °C), and the number of isotherms to be measured (two isotherms each for two separate aliquots, totaling four isotherms). Each participant was provided with one unit of RM 8852 (40 g). Participants were asked to write a brief research report describing their experimental procedures and data processing steps, and to transmit that report and the isotherms as surface excess uptake in units of millimole of CO_2_ adsorbed per gram of activated RM 8852. By-and-large the participants followed the prescribed protocol, though there were some small deviations. Further details can be found in Table 1, which lists various experimental parameters and procedures for each dataset.

**Table 1:** Experimental parameters of as-submitted datasets

Dataset	Measurement method	Gas purity (%)	Sample size (g)	Outgas condition	Sample handling/weighing and transfer
1	Manometric	CO_2_: 99.999%	1.182, 1.181	High-vac, evacuate at RT, ramped 1 K/min to 623 K, held at 623 K for 12 h	Activated ex-situ, then transferred air-free to sample holder in Ar glovebox
2	Manometric	CO_2_: 99.999% He: 99.9999%	0.659, 0.745	High-vac, ramped 1 K/min to 623 K, held at 623 K for 12 h	Activated ex-situ, transferred in air to instrument, reactivated at 623 K for 12 h using rough pump in instrument before measurement
3	Manometric	CO_2_: 99.998%	1.202, 1.199	Vac at RT for 4 h, ramped 2 K/min to 623 K, held for 40 h	Activated ex-situ, then transferred air-free to sample holder in N_2_ glovebox; once in instrument, evacuate for 2 h before measurement
4	Manometric	CO_2_: 99.9995%	~ 2	High-vac, evacuate at RT, ramped 1 K/min to 623 K, held at 623 K for at least 12 h	Activated ex-situ, transferred in air to instrument, reactivated at 573 K in instrument
5	Gravimetric	CO_2_: 99.999%	~ 0.040	High-vac (< 10^− 7^ kPa), ramped at 5 K/min to 623 K, held for 13.3 h	Activated in-situ
6	Gravimetric	CO_2_: 99.999%	0.174, 0.183	High-vac, ramped 1 K/min to 623 K, held at 623 K for 14 h	Activated in-situ
7	Manometric	CO_2_: 99.999%	~ 3	Under vac and He flow, ramped 1 K/min − 2 K/min to 623 K, hold overnight, increased vacuum to 0.034 kPa to eliminate any trace helium	Activated in-situ (final mass calculated based on % weight loss from gravimetric system of dataset #7)
	Gravimetric	CO_2_: 99.999%	0.775	Under vac and He flow, ramped 1 K/min − 2 K/min to 623 K, held for 4 h, helium vacuumed off to 2 × 10^− 5^ kPa	Activated in-situ
8	Manometric	CO_2_: 99.999%	2.231, 2.69	High-vac, 623 K for 24 h	Activated ex-situ, transferred in air/helium to instrument
9	Gravimetric	CO_2_: 99.999% He: 99.999%	0.81, 0.85	High-vac (< 10^− 5^ kPa) RT for 1 h, then ramped 1 K/min to 623 K, held for 12 h	Activated in-situ
10	Gravimetric	CO_2_: 99.999%	0.094, 0.095	High-vac, ramped 1 K/min to 623 K, held at 623 K for 18 h 40 min	Activated in-situ
11	Gravimetric	CO_2_: 99.999%	0.0675, 0.0691	High-vac (5 × 10^− 11^ kPa), ramped 1.2 K/min to 623 K, held at 623 K for 16 h	Activated in-situ
12	Manometric	CO_2_: 99.999%	1.3089, 1.147	Rough vacuum at 353 K for 1 h, ramped 1 K/min to 393 K, held 1 h, ramp 5 K/h to 623 K, held 12 h	Activated ex-situ, transferred in air to instrument
13	Gravimetric	CO_2_: 99.999%	~ 0.1	High-vac (< 1 × 10^− 5^ kPa), ramped 0.83 K/min to 423 K, then 1.67 K/min to 623 K, held at 623 K for 16 h	Activated in-situ

Dataset	Void volume/buoyancy correction	Equation of state	Temperature and stability	Pressure transducer accuracy	Blank correction^a^
1	Void volume from skeletal density (from Helium pycnometry) and mass of sample	Span and Wagner (Span and Wagner 1996)	(293.15 ± 0.1) K M^b^ = (313 ± 0.1) K	2.07 MPa and 20.7 MPa; accuracy: ±0.05% F.S.^c^	yes, Si shot
2	Void volume via He expansion	Span and Wagner	(293.15 ± 0.02) K M = (294.25 ± 0.02) K	Piezo-resistive sensor with range of 133 kPa and 13.5 MPa; accuracy: ±0.04% F.S	No
3	Void volume via He expansion at 70 kPa at 298 K, average of 10 runs	Span and Wagner	(293.15 ± 0.02) K	Below 110 kPa, accuracy ± 0.15% reading; above 110 kPa, accuracy ± 0.04% F.S. (13.3 MPa) with stability of ± 0.1%	Yes, empty sample holder
4	Void volume via He expansion at 293.15 K; average of 6 runs at different pressures	Span and Wagner	(293.15 ± 0.05) K M = (294.15 ± 0.5) K	0 kPa − 133 kPa and 0 MPa–3.33 MPa pressure transducers; accuracy: not mentioned	No
5	Buoyancy correction from ex-situ determined skeletal density and mass of sample and balance components	Span and Wagner	(293.15 ± 0.05) K	6 MPa or 20 MPa; accuracy: ± 0.04% F.S	No
6	Buoyancy correction from ex-situ determined skeletal density and mass of sample and balance components	Span and Wagner	(293.15 ± 0.1) K air bath = 298 K	Below 127 kPa, accuracy 0.12% of reading with resolution of 0.002% F.S (3.33 MPa). Above 127 kPa, accuracy ± 0.04% F.S	Yes, Si shot
7	Void volume from He expansion	Virial equation (to 2nd coefficients)	(293.15 ± 0.1) K	0 kPa − 68.9 kPa (70 Pa digital readout resolution) and 0 kPa − 689 kPa (0.69 kPa digital readout resolution)	No
	Buoyancy correction from He isotherm	Simultaneously measured CO_2_ density from mass measurement of sinker with known mass (volume)	(293 ± 1) K	0 MPa − 1.37 MPa (digital readout resolution 133 Pa) and 0 MPa − 3.45 MPa (digital readout resolution 6.89 kPa)	No
8	Void volume via He expansion	Span and Wagner	(293.10 ± 0.02) K M = (293.10 ± 0.03) K	A piezoelectric pressure transmitter with precision of 0.01% F.S	No
9	Buoyancy correction via sample volume determined from He isotherm	Span and Wagner	(293.15 ± 0.5) K	Below 133.3 kPa, resolution 1.3 Pa 133.3 kPa to 3.333 MPa, resolution 32.5 Pa Up to 16 MPa accuracy 0.1% F.S	No
10	Buoyancy correction from ex-situ determined skeletal density and mass of sample and balance components	Span and Wagner	(293.15 ± 0.02) K	0 MPa − 2.0 MPa, accuracy ± 0.04% F.S. and resolution 0.006% F.S.; 0 kPa − 10 kPa accuracy ± 0.15% reading and resolution 0.001% F.S	Yes, Si shot
11	Buoyancy correction not applied	Not used	(293.15 ± 0.1) K	0 MPa − 2.0 MPa, accuracy ± 0.02% of range (2.0 MPa)	No
12	Not mentioned	Span and Wagner	(293.15 ± 0.01) K M = (293.15 ± 0.2) K	0.1 MPa and 20 MPa; accuracy ± 0.05% F.S	Yes, empty sample holder
13	Buoyancy correction via sample volume determined from He isotherm	Lee/Kesler Generalized-correlation method (Lee and Kesler 1975)	293.15 K	0 kPa − 1.33 kPa, 0 kPa − 13.3 kPa, 0 kPa − 133 kPa, and 0 kPa − 1.333 MPa	No

^a^Blank isotherm was measured using CO_2_ at 293.15 K using either an empty sample holder or a non-adsorbing sample (Si shot)

^b^M = manifold or air bath temperature (temperature of reference volume or other components of instruments (not at the analysis site) involved in calculation of excess adsorption)

^c^F.S. = Full Scale

### As-submitted datasets

Each participant submitted at least one dataset. For clarity, a dataset is defined as being composed of four adsorption isotherms (aliquot 1–isotherm 1, aliquot 1–isotherm 2, aliquot 2–isotherm 1, and aliquot 2–isotherm 2). In total, thirteen datasets were evaluated in this analysis. In general, the isotherms were highly reproducible (see Figures S1–S3 in the Supplemental Information).

To clearly display a plot including data from all participants, the average of the four isotherms for a given dataset is shown in the figures in the text. However, there were some datasets for which averaging was not possible because the excess adsorption data were measured at different equilibrium pressure points for each of the four isotherms in the dataset. In these cases, one representative isotherm was selected for display.

### Statistical evaluation of as-submitted data

The as-submitted data were converted from excess adsorption (*n*_*ex*_) to absolute adsorption (*n*_*abs*_), using the equation (Keller and Staudt 2005; Brandani et al. 2016),

1 $${n_{abs}}=\frac{{{n_{ex}}}}{{1 - {{{\rho _{gas}}} \mathord{\left/ {\vphantom {{{\rho _{gas}}} {{\rho _{absrb}}}}} \right. \kern-0pt} {{\rho _{absrb}}}}}},$$

where *ρ*_*gas*_ is the gas density and *ρ*_*absrb*_ is the bulk density of the adsorbate, assumed here to be 0.773 g/cm^3^, the liquid density of CO_2_ at 293.15 K (20 °C).

All the isotherms of the as-submitted surface excess data were fit, collectively, to a three-parameter logistic function (Balakrishnan 1992),

2 $${n_{ex}}=\frac{\gamma }{{1+\exp [\left( { - {\text{ln}}(P)+\alpha } \right)/\beta ~]}}~,$$

where, ${n_{ex}}$ is the excess uptake (mmol/g), *P* is equilibrium pressure (MPa), and α, β, and, γ are fit parameters. The fit parameters and the associated 95% uncertainty interval were estimated using a Bayesian, Markov Chain Monte Carlo method (Possolo and Toman 2007; Gelman 2013).

### Data resubmission

Six datasets were re-evaluated after applying the evaluation methods described above. Five datasets were resubmitted. For more detailed information regarding the resubmission, see the Supplemental Information, section S4. One participant (dataset #8) was unable to explore the origin for the deviation in their dataset from the statistical mean, and this dataset was excluded from the final determination of the reference isotherm.

### Empirical reference function

To obtain an empirical reference isotherm function, the final surface excess datasets were fit to a four-parameter logistic function (Balakrishnan 1992),

3 $${n_{ex}}=\frac{d}{{{{(1+\exp [\left( { - {\text{ln}}(P)+a} \right)/b~])}^{c~~}}}}~,$$

where, *n*_*ex*_ is the excess uptake (mmol/g), *P* is equilibrium pressure (MPa), and *a, b, c*, and *d* are fit parameters, determined using the method given above. The logistic function was selected because it replicated the form of the measured isotherms. No physical significance should be associated with the function or the fit parameters.

## Results and discussions

**Fig. 1:**
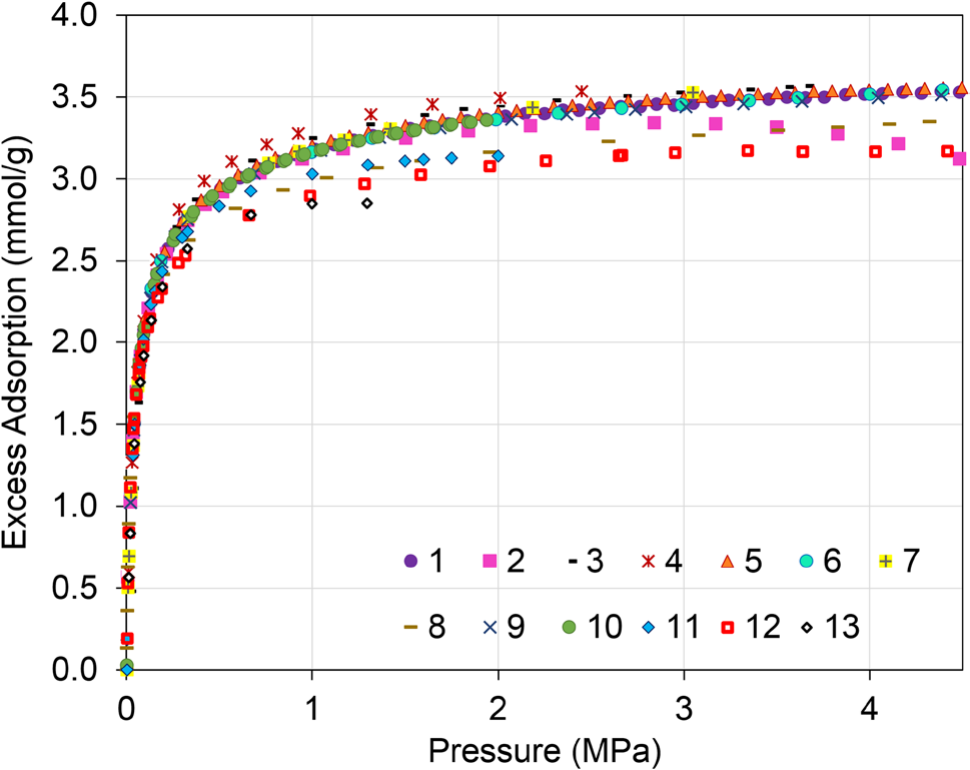
As-submitted surface excess CO_2_ adsorption isotherms at 293.15 K (20 °C) for RM 8852 (For low-pressure data and semi-log-scale see Figures S6 and S7.)

The thirteen as-submitted datasets are shown in Fig. 1. Seven datasets report similar uptakes (#1, 3, 5, 6, 7, 9, and 10). One dataset (#4) shows uptake slightly above this cluster, while five datasets have lower uptake (#2, 8, 11, 12, and 13). One dataset (#2) exhibits a noticeably different pressure dependence. To evaluate more rigorously the quality and comparability of the as-submitted data, the as-submitted excess adsorption data isotherms were converted to absolute adsorption. The surface excess isotherms were also fit to Eq. 2. When plotted as absolute adsorption, it is expected that an isotherm should monotonically increase as a function of pressure. All the datasets exhibit the expected trend, except for one (#2). To assess statistical variability, the as-submitted excess adsorption data were fitted, collectively, to Eq. (2). Six of the thirteen datasets (#2, 4, 8, 11, 12, 13) were outside the expanded uncertainty interval of the best-fit to the collective dataset (see Figure S5).

Further evaluation of the as-submitted data and the associated research reports identified reasons for the observed variation in the outlying surface excess datasets, which included the following: incomplete activation of the sample (#12) and inaccurate determination of sample mass (#4, 12); inaccurate sample skeletal volume determination (#11, 13); lack of a buoyancy correction, when using a gravimetric system (#11); improper choice or application of an equation of state for CO_2_ (#13); and the need for a blank correction (#2, 11, 13).

Insufficient sample activation (e.g. outgassing with just a rotary pump) led to lower adsorbed amounts at higher pressures, in line with previous reports in the literature (Gensterblum et al. 2009). Mass measurement errors can result from incomplete sample activation, sample rewetting following ex-situ activation, or measuring the mass of empty sample holder and sample under different physical conditions. As the uptake is reported as “per gram of activated adsorbent,” inaccurate sample mass determination affects the uptake proportionally.

The skeletal volume of the sample, which is needed for void volume determination and buoyancy correction, affects the calculation of surface excess uptake. For RM 8852, a skeletal density value of ≈2.36 g/cm^3^ should be used as a guide to determine the sample volume.

While the effect is minor for low-pressure isotherms, the lack of a buoyancy correction can significantly impact high-pressure data when using gravimetric instruments. The magnitude and direction of the discrepancy of the uncorrected data depends on the buoyancy force acting on the sample, which depends on the balance set-up of the instrument.

For adsorption measurements with fluids near the critical region, where the compressibility of the gas is significant at high pressures (such as for CO_2_ at room temperature), it is also important to consistently use a critically evaluated equation of state, e.g. for CO_2_, the Span–Wagner equation (Span and Wagner 1996). In addition, the appreciable compressibility of CO_2_ at higher pressures at 293.15 K (20 °C) coupled with other experimental limitations, such as insufficient temperature stability and homogeneity in key areas of the adsorption apparatus, can lead to additional uncertainties that can be accounted for by a blank adsorption experiment.

**Fig. 2:**
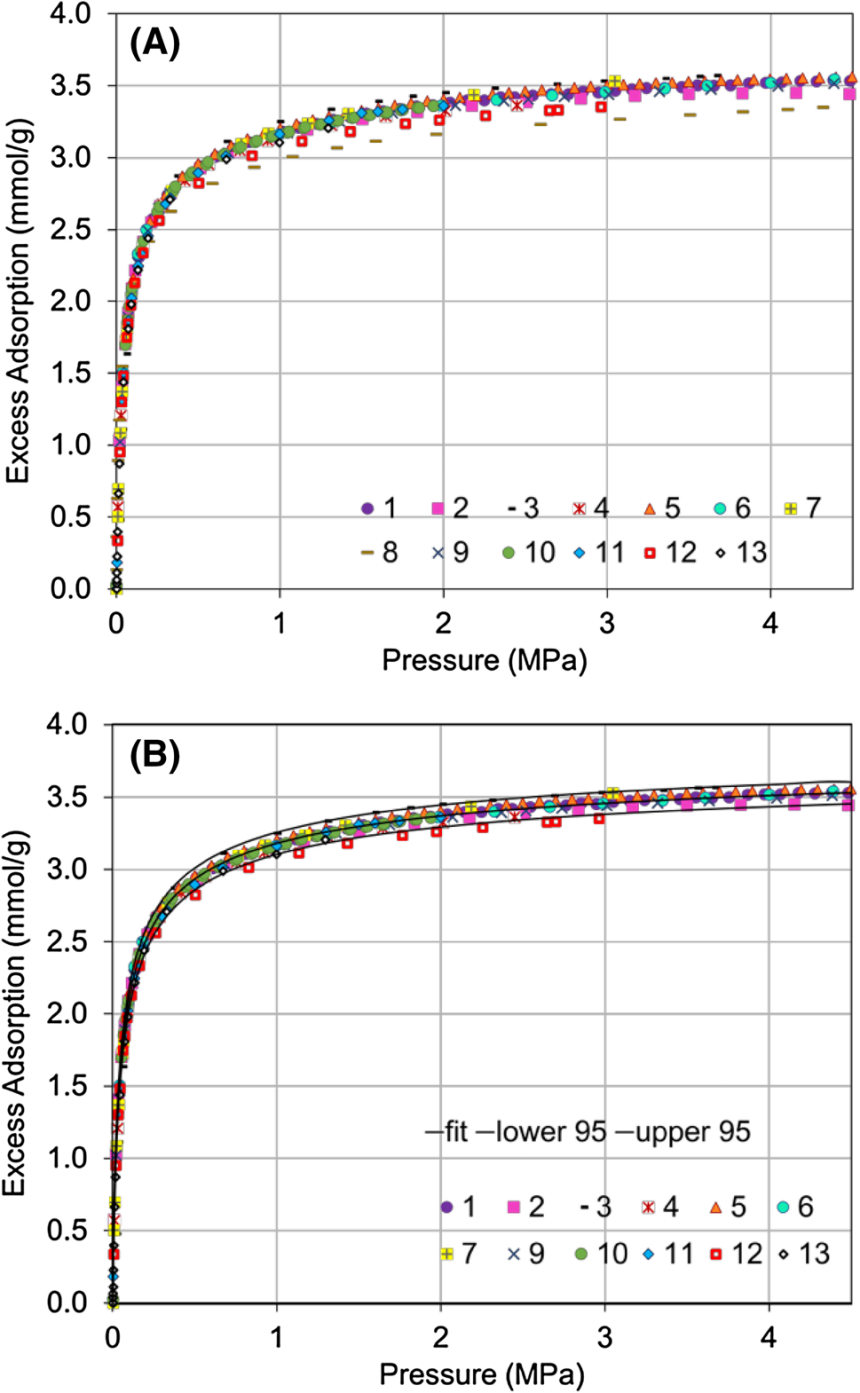
**a** Final surface excess CO_2_ adsorption isotherms at 293.15 K (20 °C).** b** Best fit to the isotherm data and 95% uncertainty interval. (For low-pressure data and logarithmic scales see Figures S8–S10. Residuals of the fit are shown in Figure S11.)

After datasets #2, 4, 11, 12, and 13 were re-submitted, the final surface excess datasets for CO_2_ adsorption on RM 8852 were obtained. As shown in Fig. 2A, these final datasets are in good agreement. An empirical surface excess reference function was determined by optimizing the fit of Eq. 3 to the final datasets and is shown in Fig. 2B. The parameters for this empirical reference isotherm are *a* = −6.22 (0.08), *b* = 1.97 (0.01), *c* = 4.73 (0.21), and *d* = 3.87 (0.01). (The standard error for each fit parameter is shown in parenthesis.) This function is predictive from 1 kPa up to 4.5 MPa and has expanded uncertainty, *U*(*k* = 2), for the excess uptake of approximately 0.075 mmol/g over the full pressure range. The final datasets and the reference isotherm with its 95% uncertainty interval are available through the NIST Database of Novel and Emerging Adsorbent Materials (Siderius et al. 2014, 2018).

## Recommendations

The protocol to use RM 8852 and the associated reference CO_2_ adsorption isotherm at 293.15 K (20 °C) are provided in Section S1 in the Supplemental Information. In addition, based on this work, the following recommendations for measuring of this high-pressure CO_2_ adsorption isotherm are offered:

- Sample activation—Sufficiently complete sample activation is crucial. Typically, for microporous materials, such as ZSM-5, this will involve outgassing at high-temperature and vacuum, though other approaches may be adequate for achieving comparable activation (Thommes et al. 2015). RM 8852 requires special handling to ensure the sample is activated completely. It must be activated at 623 K (350 °C) for at least 12 h under high vacuum (≤ 1 cPa) to realize the reported reference isotherm. If the sample is activated ex-situ, exposure to air and moisture must be avoided to obtain the correct sample mass.
- Sample volume determination—Proper determination of sample volume is needed both for buoyancy correction in a gravimetric system, as well as the void volume determination in a manometric system (International Organization for Standardization 2014; Belmabkhout et al. 2004). If required in data analysis, a skeletal density of ≈2.36 g/cm^3^ should be used for RM 8852.
- Buoyancy correction/void volume correction—A buoyancy correction must be applied when using a gravimetric system. Although less important at low-pressures, buoyancy effects are significant for high-pressure measurements and cannot be overlooked (Nguyen et al. 2017; Rouquerol et al. 1999). This is analogous to the use of void volume in a manometric instrument to determine surface excess uptake, and the effect of using the wrong volume also becomes more significant with increasing pressure.
- Equation of state—In general, identify the equation of state used to calculate fluid density, and use critically evaluated equations, such as those contained in the NIST Reference Fluid Thermodynamic and Transport Properties Database (REFPROP) (Lemmon et al. 2013). The Span and Wagner equation of state should be used for CO_2_ adsorption at 293.15 K (20 °C).
- *T, P*, and *m*—Ensure good control and measurement of temperature (*T*), pressure (*P*), and sample mass (*m*), as these are important in accurate determination of the uptake (Broom and Webb 2017; Belmabkhout et al. 2004).
- Blank correction—A blank run subtraction should be performed whenever possible, as it corrects for small uncompensated transducer nonlinearities, effects of temperature heterogeneities coupled with the compressibility of the adsorptive, and other experimental effects (Nguyen et al. 2017).

## Conclusions and outlook

This work presents an empirical reference isotherm function for high-pressure CO_2_ adsorption on NIST RM 8852. It was demonstrated that even when using diverse instruments—gravimetric, manometric, commercial, custom-built—it is possible to obtain consistent surface excess isotherms when attention is paid to sample handling and data processing. The reference isotherm function and the associated reference material provide, for the first time, a means for laboratories to test and validate high-pressure adsorption equipment and measurements. This work should also prove to be a useful resource for those learning to make adsorption measurements.

This ILS was unique in that the as-submitted datasets were evaluated and in collaboration with participating laboratories, the causes for differences among datasets were identified, and laboratories were given the opportunity to reprocess data or remeasure adsorption isotherms before the reference isotherm function was derived.

In a forthcoming exercise, a new ILS will be undertaken, for high-pressure adsorption of methane on NIST Reference Material RM 8850 (zeolite Y). The methane ILS is being organized through Technical Working Group 39 of the Versailles Project on Advanced Materials and Standards (VAMAS).^2^ Replication of the reference data generated in this CO_2_ ILS will be a requirement for participation in the upcoming methane exercise. The methane ILS will provide another unique dataset that will aid in the proper use of high-pressure adsorption equipment.

## Electronic supplementary material

Below is the link to the electronic supplementary material.

Supplementary material 1 (DOCX 2136 KB)

## Footnotes

1 http://www.nist.gov/mml/fact.

2 http://www.vamas.org/twa39/.
