# Supplementary material for: A reference high-pressure CO2 adsorption isotherm for ammonium ZSM-5 zeolite: results of an interlaboratory study
Source: Adsorption (Boston). 2018 Jul 26;24(6):531–9. doi: 10.1007/s10450-018-9958-x (PMC6417222; doi:10.1007/s10450-018-9958-x)
Supplement: Supplementary file 1 — Supplementary material 1 (DOCX 2136 KB) [file 10450_2018_9958_MOESM1_ESM.docx]

**Supplemental Information to accompany:**

**“A Reference High-Pressure CO_2_ Adsorption Isotherm for Ammonium ZSM-5 Zeolite: Results of an Interlaboratory Study”**

H.G.T. Nguyen,^1^ L. Espinal,^1^ R.D. van Zee,^1^ M. Thommes,^1,2^ B. Toman,^1^ M.S.L. Hudson,^1^ E. Mangano,^3^ S. Brandani,^3^ D.P. Broom,^4^ M.J. Benham,^4^ K. Cychosz,^2^ P. Bertier,^5^ F. Yang,^5^ B. M. Krooss,^5^ R.L. Siegelman,^6^ M. Hakuman,^7^ K. Nakai,^7^ A. Ebner,^8^ L. Erden,^8^ J. A. Ritter,^8^

A. Moran,^9^ O. Talu,^9^ Y. Huang,^10^ K.S. Walton,^10^ P. Billemont,^11^ G. De Weireld^11^

^1^National Institute of Standards and Technology, Gaithersburg MD, USA

^2^Quantachrome Instruments, Boynton Beach FL, USA

^3^University of Edinburgh, Edinburgh, GBR

^4^Hiden Isochema Ltd, Warrington, GBR

^5^RWTH Aachen University, Aachen, DEU

^6^University of California, Berkeley, Berkeley CA, USA

^7^MicrotracBEL, Suminoe-ku, Osaka, JPN

^8^University of South Carolina, Columbia SC, USA

^9^Cleveland State University, Cleveland OH, USA

^10^Georgia Institute of Technology, Atlanta GA, USA

^11^University of Mons, Mons, BEL

**Table of Contents**

S1. Protocol to use RM 8852 and its associated CO_2_ reference isotherm

S2. Determination of the number of datasets

S3. As-submitted isotherms (datasets #1-13)

S4. Isotherms for which re-evaluation was suggested to the participant (datasets #2, 8, 4, 11, 12, and 13)

S5. Analysis of as-submitted datasets.

S6. Comparative plots of as-submitted and final isotherms

S7. Residuals from the empirical reference function

**S1. Protocol to use RM 8852 and carbon dioxide reference isotherm. (Recommended practices for realizing the reference isotherm are provided in the text.)**

Materials: The adsorbent is NIST reference material [RM 8852](https://www-s.nist.gov/srmors/quickSearch.cfm?source2=tables&srm=8852&go=Go) (ammonium ZSM-5 zeolite). The sample mass to be used should be optimized to achieve the best signal-to-noise ratio, according to the experimental parameters of the instrument being used. The adsorptive, carbon dioxide, should have a purity of not less than 99.999 %.

Sample pretreatment: The zeolite should be outgassed using a turbomolecular pump and slowly heated to 623 K (1 degree per minute, nominally) and held at that temperature for at least 12 hours under high vacuum (<10^-5^ kPa) with continuous pumping. If the outgassing is performed in a separate manifold, prevent contact with air when transferring to the analysis instrument.

Measurement of adsorption isotherms: Carbon dioxide adsorption isotherms should be measured at 293.15 K over a pressure range of 1 kPa to 4.5 MPa. The same measurement is also to be done without the adsorbent to perform a “blank correction.” This blank should be subtracted from the adsorption isotherm.^[[1]](#footnote-1)^ The Span and Wagner^[[2]](#footnote-2)^ equation of state is to be used to determine the density/compressibility of the gas for the determination of surface excess uptake. RM 8852 has a skeletal density of ≈2.36 g/cm^3^, which is to be used when performing buoyancy correction for gravimetric technique or void volume determination for volumetric technique.

Comparison to the empirical reference isotherm function: Measured isotherms should fall within the 95% uncertainty interval of the reference isotherm function,

$$n_{ex}=\frac{d}{({1+\exp[\left( -ln(P)+a \right)/b ])}^{c}} ,$$

where *n_ex_* is the excess uptake (mmol/g), *P* is equilibrium pressure (MPa), and *a*, *b*, *c*, and *d* are fit parameters with values of *a* = -6.22 (0.08), *b* = 1.97 (0.01), *c* = 4.73 (0.21), and *d* = 3.87 (0.01).

**S2. Determination of the number of datasets:**

There were a total of 13 datasets submitted. One participant used a manometric and gravimetric system to cover different pressure ranges (low and high, respectively) in order to generate isotherms over the full pressure range (dataset #7). Another participant performed measurements over the full pressure range with both a gravimetric and a manometric instrument (datasets #1, 6). This participant also measured an isotherm using a second gravimetric instrument, but only up to 2.0 MPa (dataset #10). Thus, this participant submitted three unique datasets, and each was processed individually. Another participant also submitted three datasets, but used three identical instruments; hence only one representative dataset was chosen for analysis (dataset #5).

**S3. As-submitted isotherms (datasets #1-13)**


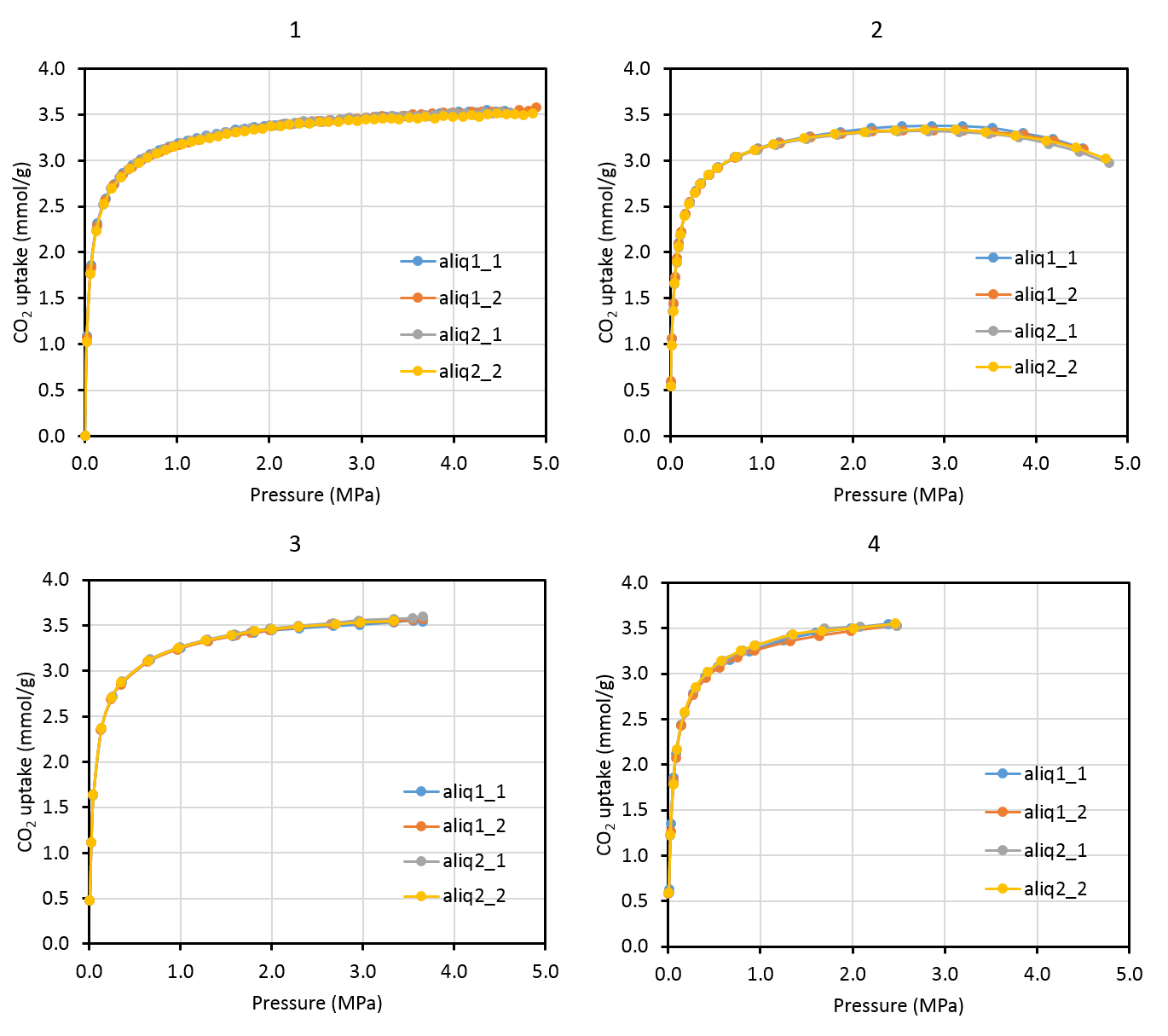


Figure S1. As-submitted excess CO_2_ adsorption isotherms at 293.15 K for RM 8852 for datasets 1-4.


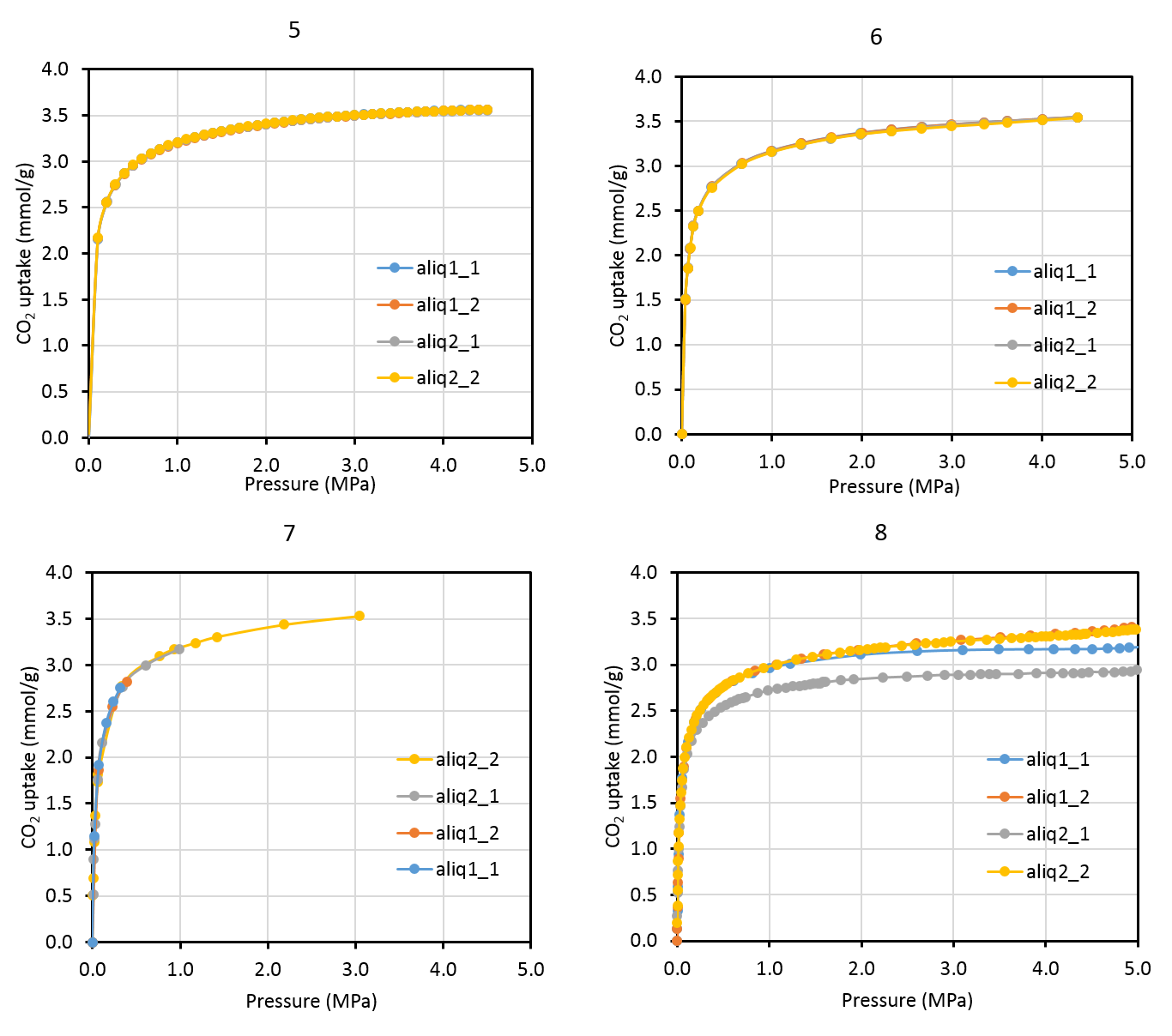


Figure S2. As-submitted excess CO_2_ adsorption isotherms at 293.15 K for RM 8852 for datasets 5-8.


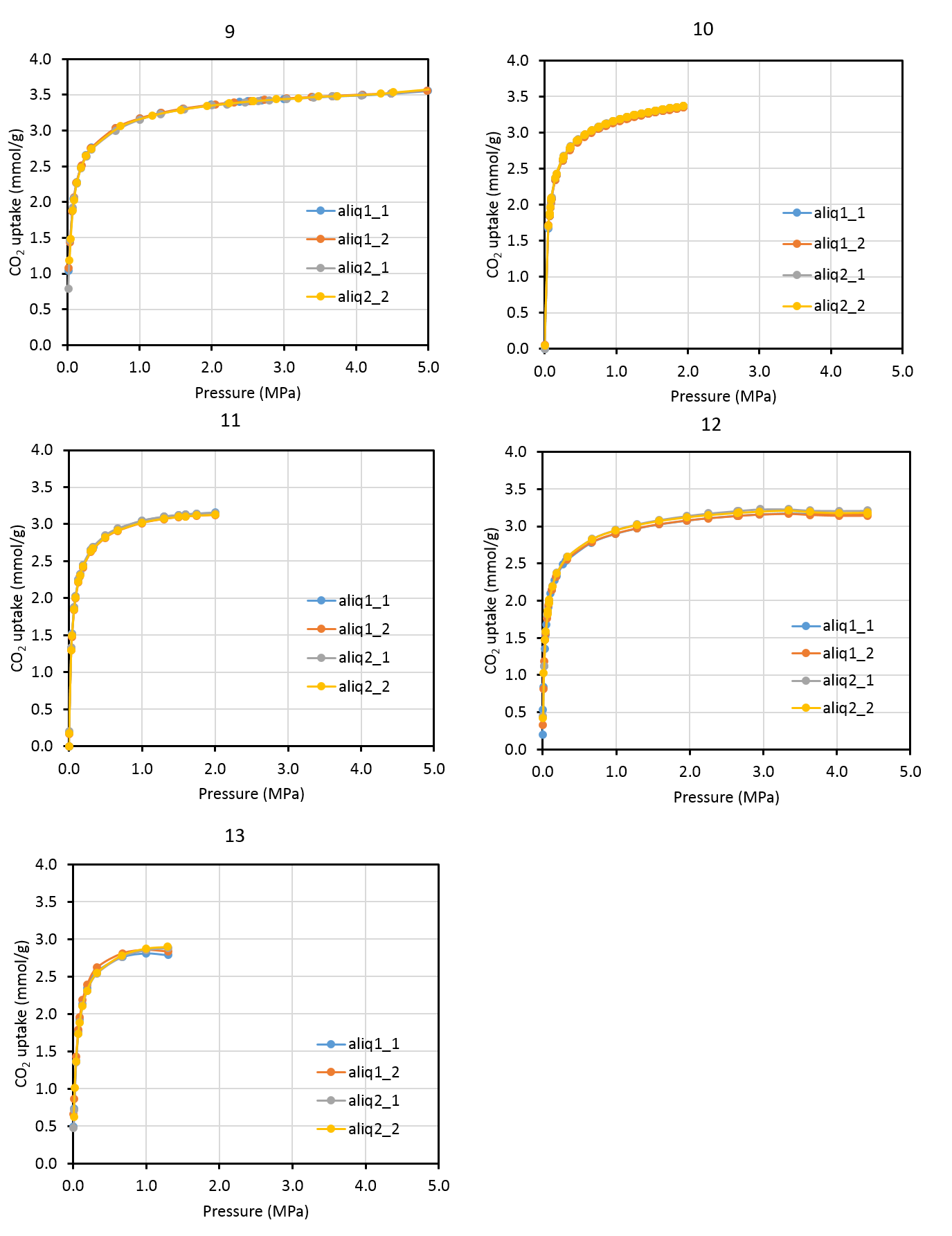


Figure S3. As-submitted excess CO_2_ adsorption isotherms at 293.15 K for RM 8852 for datasets
9-13.

**S4. Isotherms for which re-evaluation was suggested to the participant (datasets #2, 8, 4, 11, 12, and 13)**

Table S1. Details of re-evaluation/resubmission

| **Dataset** | **Changes made for resubmission** |
| --- | --- |
| 2 | Applied blank correction* |
| 4 | Corrected mass measurement of low-pressure isotherm that was used to normalize the high-pressure isotherm |
| 8 | Unable to perform additional measurements. Isotherms were outside the 95% confidence interval both as-submitted and in comparison to final datasets, and thus were excluded from determination of the reference isotherm. |
| 11 | Resubmission 1: applied buoyancy correction and applied blank correction.  Resubmission 2: reapplied buoyancy correction using an appropriate sample skeletal density for RM 8852. |
| 12 | Performed sample activation under high-vacuum using a turbomolecular pump. |
| 13 | Performed He buoyancy measurements at elevated temperature and applied blank correction. |

***** An adsorption isotherm without the adsorbent in the instrument and subtract this “background isotherm” from the adsorption isotherm.


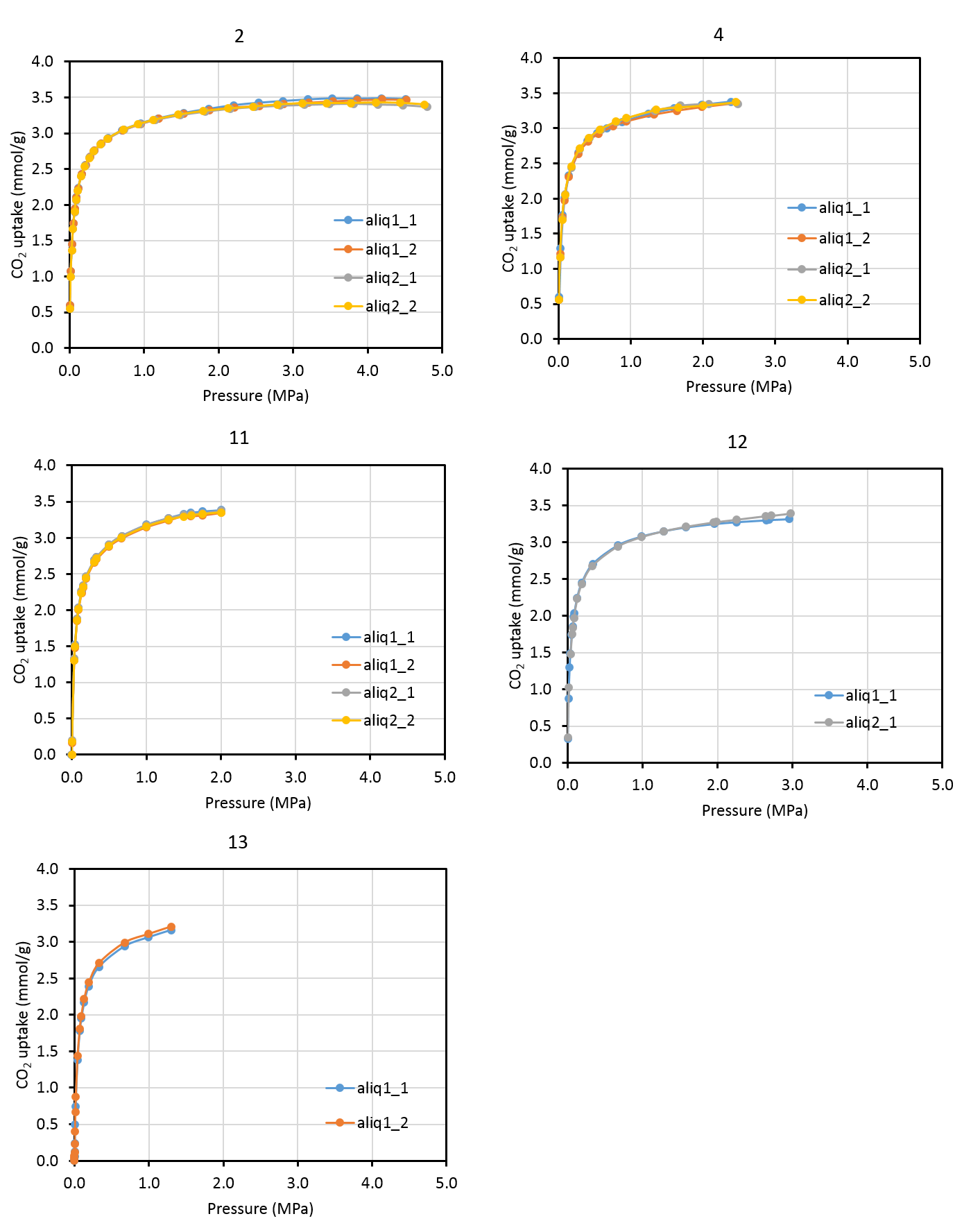


Figure S4. Resubmitted excess CO_2_ adsorption isotherms at 293.15 K for RM 8852 for datasets 2, 4, 11-13.

**S5. Analysis of as-submitted datasets**

**
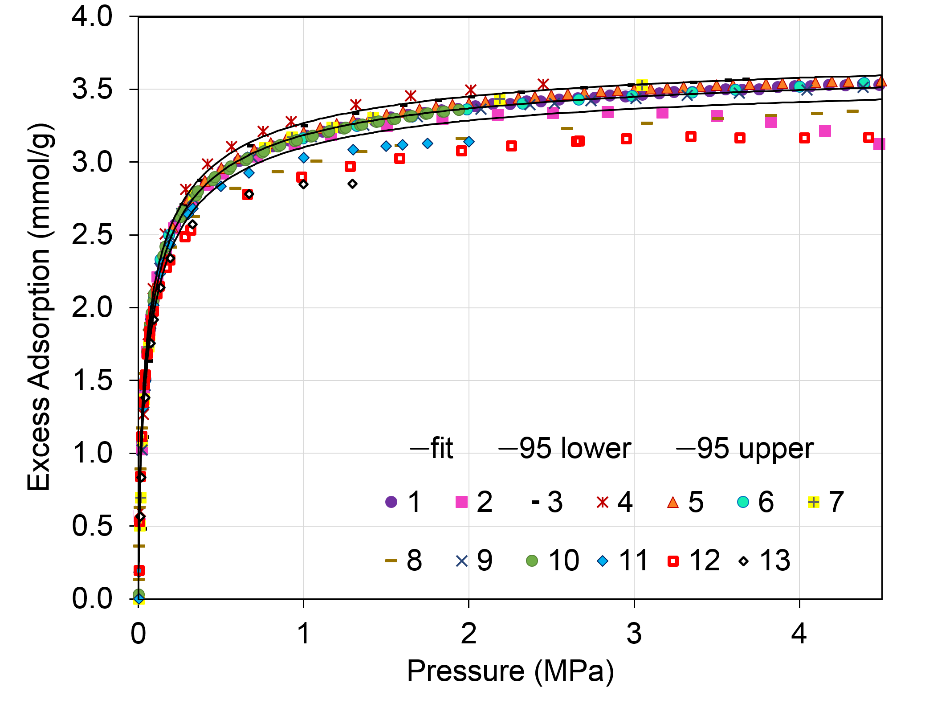
**

Figure S5. As-submitted surface excess adsorption isotherms with the best fit and 95% uncertainty intervals. The fit parameters and the associated 95 % uncertainty interval were estimated using a Bayesian, Markov Chain Monte Carlo method,^[[3]](#footnote-3)^ yielding *α* = −2.658(0.006), *β* = 1.519(0.010), and *γ* = 3.743(0.006). The expanded uncertainty, *U*(*k*=2), for the excess uptake was approximately 0.084 mmol/g over the full pressure range. The logistic function was selected because it replicated the form of the measured isotherms. No physical significance should be associated with the function or the fit parameters.

**S6. Comparative plots of as-submitted and final isotherms**


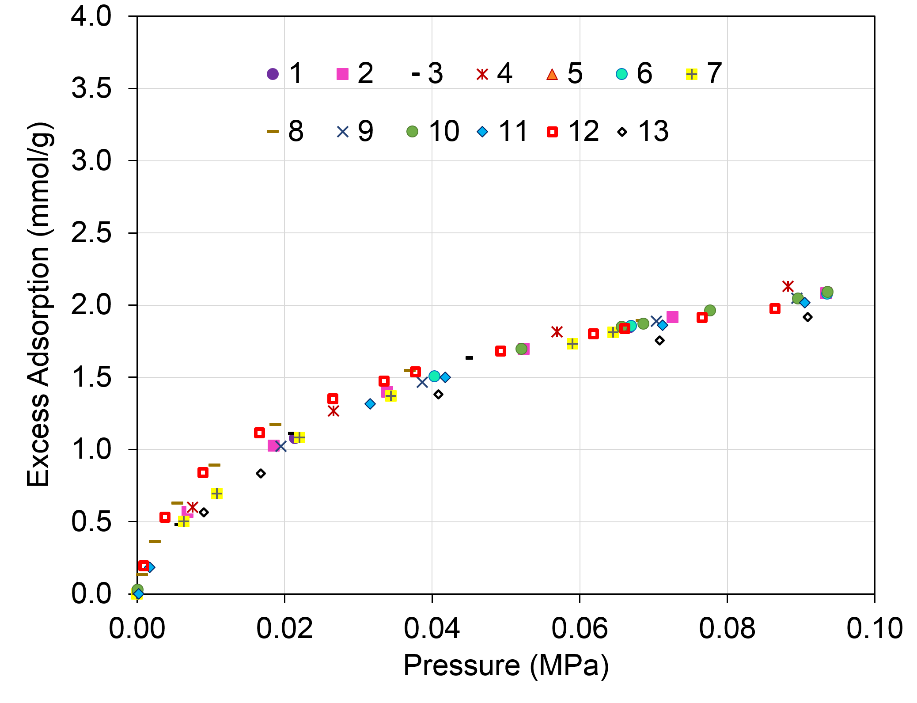


Figure S6. As-submitted excess CO_2_ adsorption isotherms at 293.15 K for RM 8852 shown to 0.1 MPa.


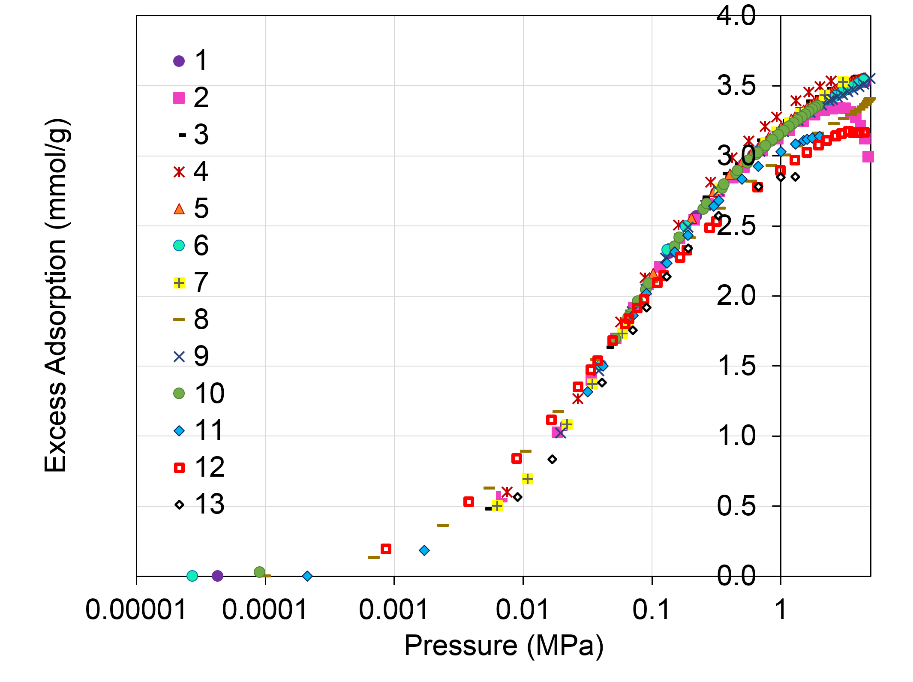


Figure S7. As-submitted excess CO_2_ adsorption isotherms at 293.15 K for RM 8852 shown in a semi‑log plot.


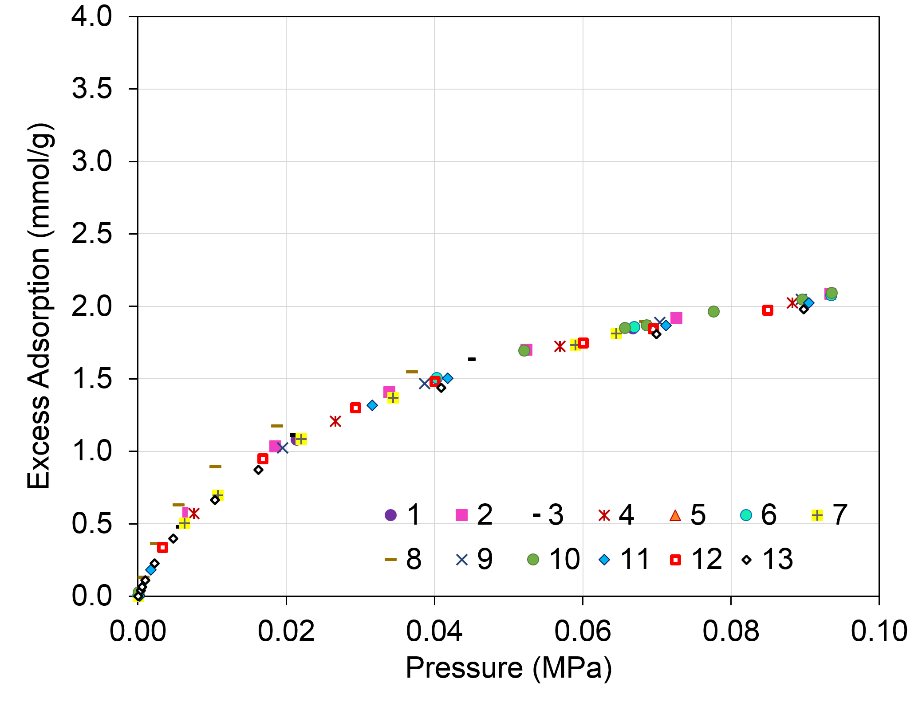


Figure S8. Final excess CO_2_ adsorption isotherms at 293.15 K for RM 8852 shown to 0.1 MPa.


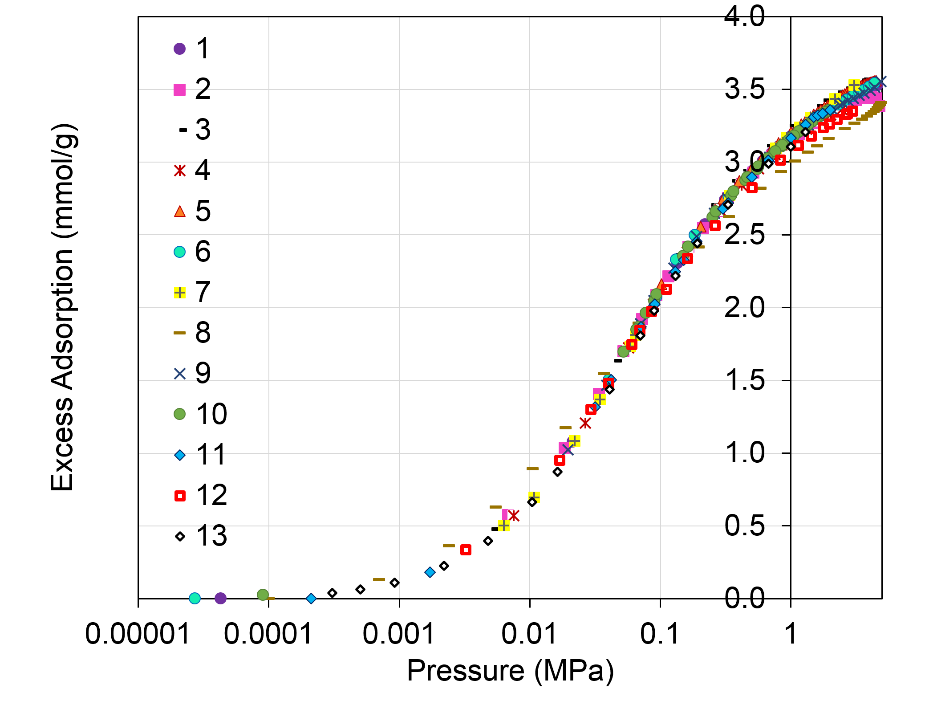


Figure S9. Final excess CO_2_ adsorption isotherms at 293.15 K for RM 8852 shown in a semi‑log plot.


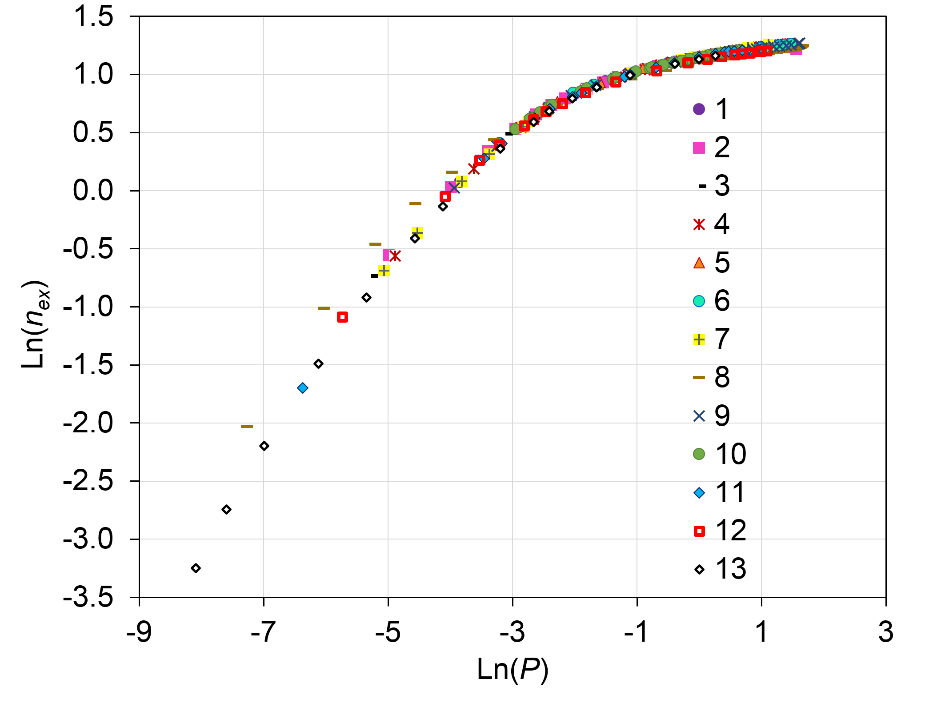


Figure S10. Final excess CO_2_ adsorption isotherms at 293.15 K for RM 8852 plotted as Ln(*n_ex_*) vs. Ln(*P*), where *n_ex_* is uptake in mmol/g and *P* is pressure in MPa.

**S7. Residuals from the empirical reference function.**

**
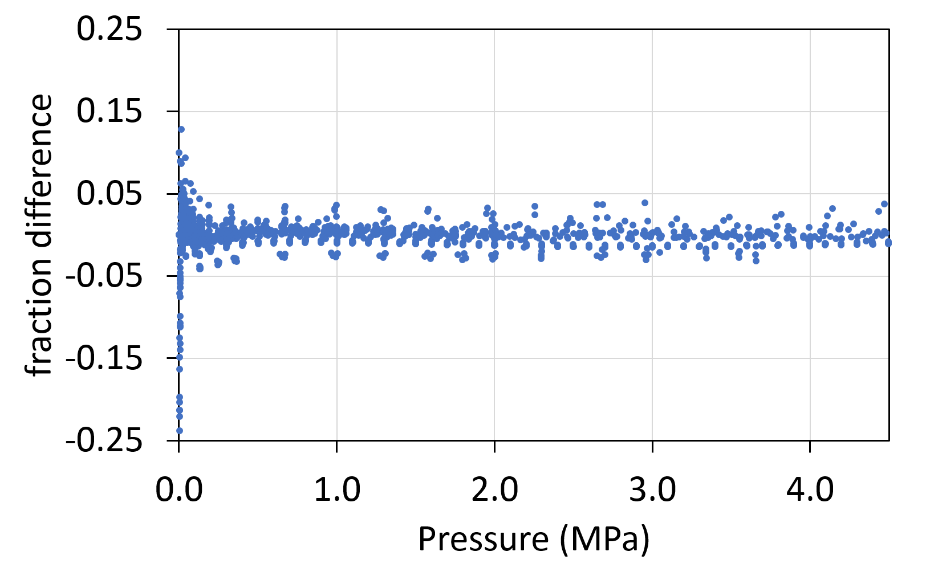
**

Figure S11. Fractional difference of the final excess CO_2_ adsorption isotherms at 293.15 K for RM 8852 from the empirical reference function.

1. H. G. T. Nguyen, J. C. Horn, M. Thommes, R. D. van Zee, L. Espinal, Experimental Aspects of Buoyancy Correction in Measuring Reliable High-pressure Excess Adsorption Isotherms Using the Gravimetric Method, *Meas. Sci. Technol.* **2017**, *28*, 125802. [↑](#footnote-ref-1)
2. R. **Span**, W. **Wagner**, A New Equation of State for **Carbon Dioxide** Covering the Fluid Region from Triple-Point Temperature to 1100 K at Pressures up to 800 MPa, *J. Phys. Chem. Ref. Data*, **1996**, *25*, 1509-1597. [↑](#footnote-ref-2)
3. (a) Possolo, A.; Toman, B., Assessment of measurement uncertainty via observation equations. *Metrologia* **2007**, *44*, 464-475; (b) Gelman, A. C., J; Stern, H; Dunson, D; Vehtari, A; Rubin, D., Bayesian Data Analysis. Chapman & Hall: Boca Raton, 2013. [↑](#footnote-ref-3)
